# Evaluation of a call center to assess post-discharge maternal and early neonatal outcomes of facility-based childbirth in Uttar Pradesh, India

**DOI:** 10.1371/journal.pone.0207987

**Published:** 2018-11-27

**Authors:** Jonathon D. Gass, Katherine Semrau, Fatima Sana, Anup Mankar, Vinay Pratap Singh, Jennifer Fisher-Bowman, Brandon J. Neal, Danielle E. Tuller, Bharath Kumar, Stuart Lipsitz, Narender Sharma, Bhala Kodkany, Vishwajeet Kumar, Atul Gawande, Lisa R. Hirschhorn

**Affiliations:** 1 Ariadne Labs, Brigham & Women’s Hospital and Harvard T.H. Chan School of Public Health, Boston, Massachusetts, United States of America; 2 Department of Medicine, Harvard Medical School, Boston, Massachusetts, United States of America; 3 Population Services International- India, New Delhi, India; 4 Jawaharlal Nehru Medical College, Belgaum, Karnataka, India; 5 Community Empowerment Lab, Lucknow, Uttar Pradesh, India; 6 Northwestern University Feinberg School of Medicine, Chicago, Illinois, United States of America; National Perinatal Epidemiology Unit, University of Oxford, UNITED KINGDOM

## Abstract

**Background:**

Maternal and neonatal outcomes in the immediate post-delivery period are critical indicators of quality of care. Data on childbirth outcomes in low-income settings usually require home visits, which can be constrained by cost and access. We report on the use of a call center to measure post-discharge outcomes within a multi-site improvement study of facility-based childbirth in Uttar Pradesh, India.

**Methods:**

Of women delivering at study sites eligible for inclusion, 97.9% (n = 157,689) consented to follow-up. All consenting women delivering at study facilities were eligible to receive a phone call between days eight and 42 post-partum to obtain outcomes for the seven-day period after birth. Women unable to be contacted via phone were visited at home. Outcomes, including maternal and early neonatal mortality and maternal morbidity, were ascertained using a standardized script developed from validated survey questions. Data Quality Assurance (DQA) included accuracy (double coding of calls) and validity (consistency between two calls to the same household). Regression models were used to identify factors associated with inconsistency.

**Findings:**

Over 23 months, outcomes were obtained by the call center for 98.0% (154,494/157,689) consenting women and their neonates. 87.9% of call center-obtained outcomes were captured by phone call alone and 12.1% required the assistance of a field worker. An additional 1.7% were obtained only by a field worker, 0.3% were lost-to-follow-up, and only 0.1% retracted consent. The call center captured outcomes with a median of 1 call (IQR 1–2). DQA found 98.0% accuracy; data validation demonstrated 93.7% consistency between the first and second call. In a regression model, significant predictors of inconsistency included cases with adverse outcomes (p<0.001), and different respondents on the first and validation call (p<0.001).

**Conclusions:**

In areas with widespread mobile cell phone access and coverage, a call center is a viable and efficient approach for measurement of post-discharge childbirth outcomes.

## Background

Maternal and neonatal outcomes in the immediate post-delivery period are critical indicators of quality of care [1]. Traditionally, near real-time measurement of outcomes has targeted in-facility events [2, 3]; however, the risk of death for both mother and baby remains alarmingly high in the seven days following delivery [4, 5]. Home visits, population health surveys, and medical record review have, historically, been the standards for measuring post-delivery outcomes in resource-limited settings [6, 7]. Home visits can be resource-intensive and limited by local constraints, including access and safety. Population health surveys are only conducted at set intervals, are typically powered to estimate events at a national or large subnational level, and include recall over long time frames. Facility-based record review can only document outcomes that occur prior to discharge. A feasible, timely, and less costly approach to collecting post-discharge outcomes is clearly needed.

Between 2000 and 2010, mobile phone use increased by 1500% in low- and middle-income countries [8]. As of 2010, 77% of global mobile subscriptions were in developing countries, where a median of 84% of individuals own cell phones [8, 9]. As mobile phone coverage increases globally, patient follow-up strategies need to capitalize on this rapid growth.

Call centers have a history of use in public health, primarily for measurement of population health or disease management in high-income countries [10–12]. In low-income countries, call centers have been used to track health status after hospitalization [13, 14], measure patient satisfaction following healthcare delivery [15], and conduct high-frequency, mobile-phone panel surveys to facilitate livelihood monitoring [16]. A few studies have demonstrated the feasibility of computer assisted telephone interviewing in developing countries [17–19]; however, there are limited published data on the quality and validity of telephonic surveillance in these settings.

We conducted the BetterBirth Trial [20], a matched-paired, cluster randomized controlled trial (RCT), testing the impact of a coaching-based implementation of the WHO Safe Childbirth Checklist on: (i) quality of care in facility-based deliveries in Uttar Pradesh, India and (ii) maternal and early neonatal morbidity and mortality in the early post-partum period [21]. A sample of 157,689 mothers and their babies across 120 sites were followed to assess health outcomes [22]. The results of this study, published elsewhere, demonstrate higher adherence to essential birth practices overall at intervention facilities compared to control sites. However, maternal and perinatal mortality and maternal morbidity did not significantly differ between the two groups (overall perinatal mortality rate: 48 deaths/1,000 births; maternal mortality ratio: 95 deaths/100,000 livebirths). The scale of follow-up during this trial across a large geographic region necessitated the establishment of a call center to ascertain patient-reported maternal and early neonatal post-discharge health outcomes via a brief telephonic interview. We developed Data Quality Assurance (DQA) protocols [23] to measure and ensure the accuracy of data collection as well as to validate the call center model in this setting.

This evaluation aimed to measure the effectiveness, efficiency, cost, and accuracy of utilizing a call center to capture patient-reported post-delivery outcomes in Uttar Pradesh, India. Of note, patient-reported outcomes were not clinically validated with clinical records or assessments as part of this study.

## Methods

### Call center design

#### Outcomes assessment questionnaire

To measure patient-reported post-delivery outcomes, a health outcomes questionnaire was developed, including a standardized script and validated questions from previous population surveys [24–27]. We utilized the WHO near-miss criteria as a basis for survey development. In order to make it feasible to ask the questions over the phone, we adapted questions as appropriate (Table 1). The questionnaire was translated from English to Hindi, back-translated to English, and pilot tested to ensure accuracy.

**Table 1:** Outcomes assessed for the 7-day period post-delivery [28].

Outcome of interest	WHO Near-Miss Criteria [28, 29]	BetterBirth Questionnaire
**Maternal and early neonatal mortality**		
Mother vital status (alive/dead)		How is the mother’s health? If mother was found to have died after discharge, when did the mother die?
Baby vital status (alive/dead)		How is the baby’s health? If baby was found to have died after discharge, when did the baby die?
**Maternal morbidity**		
Seizure (no/yes): a measure of severe preeclampsia	Uncontrollable fit/total paralysis	Did you have a fit/seizure during or after delivery at any time up until now? [25–27, 30, 31]
Fever with vaginal discharge (no/yes): a potential marker of sepsis or severe systemic infection	Sepsis or severe systemic infection	Did you have a fever during or after delivery at any time up until now? If yes, did this fever come with smelly vaginal discharge? [25, 27, 30]
Stroke (paralysis) (no/yes): a measure of eclampsia	Stroke is a neurological deficit of cerebrovascular cause that persists beyond 24 hours or is interrupted by death within 24 hours.	Did you have a stroke (paralysis) during or after delivery at any time up until now? [24]
Excessive bleeding (no/yes): a measure of severe post-partum hemorrhage	Severe postpartum hemorrhage	Did you have a lot of bleeding during or after delivery at any time up until now? If yes, did the blood wet your clothes, the bed, or the floor? [25, 26, 30–33]
Loss of consciousness for >1 hour (no/yes): a measure of neurological dysfunction, metabolic coma, or otherwise prolonged lack of responsiveness to external stimuli	Loss of consciousness is a profound alteration of mental state that involves complete or near-complete lack of responsiveness to external stimuli.* It is defined as a Coma Glasgow Scale <10 (moderate or severe coma).	Did you remain unconscious for more than 1 hour during or after delivery at any time up until now? [34]
**Critical Interventions**		
Receive a blood transfusion (no/yes)	Transfusion of 5 units red cell transfusion	Did you receive a blood transfusion during or after delivery at any time up until now?
Operation to remove uterus or womb (no/yes)	In the maternal near-miss context, surgical removal of the uterus following infection or hemorrhage	Did you have an operation to remove your uterus or womb at any time after delivery up until now?
**Mother or baby return to facility**		
Mother return to facility for a health problem (no/yes)		After you left the facility to go home, did you have to go back to a health care facility because of a problem?
Baby return to facility for a health problem (no/yes)		After the baby left the facility to go home, did anyone have to bring your baby back to a health care facility because of a problem?

* Although the WHO definition for prolonged unconsciousness stipulates any loss of consciousness lasting more than 12 hours, there is evidence that reduced oxygen saturation during loss of consciousness for greater than one hour is associated with maternal death [34].

### Call center data capture system and dashboard

A data collection system and dashboard were designed to capture and store patient contact information and manage call center follow-up. Data collectors extracted consenting mothers’ contact information from facilities’ registers and input them into a tablet-based application twice per week. The contact information was sent to the call center’s dashboard via a secure cloud-based server, where cases remained in queue until eligible for follow-up. Additionally, the dashboard selected cases for DQA.

### Outcomes assessment protocol

**Fig 1:**
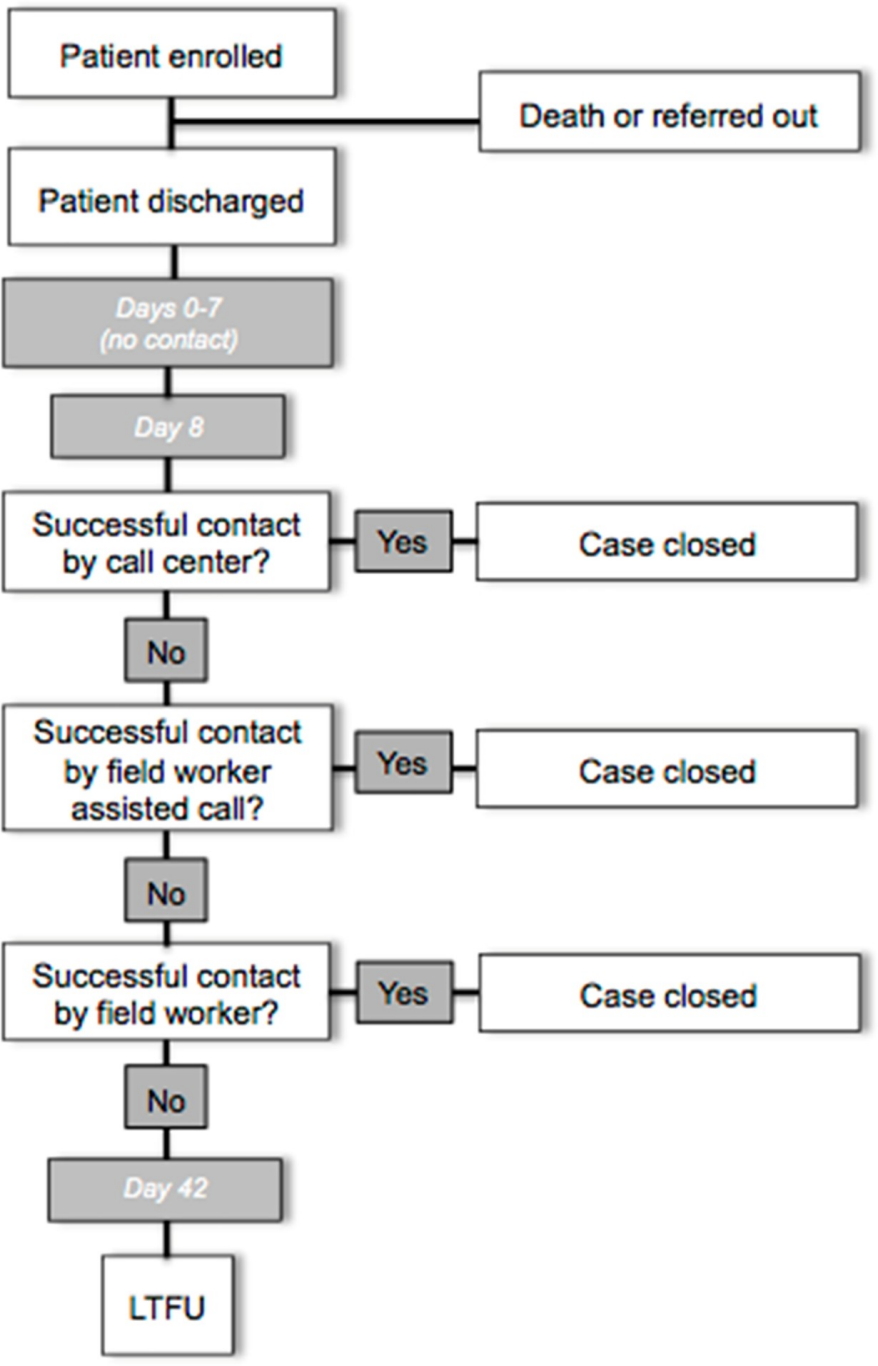
Outcomes data collection process diagram.

The Betterbirth Trial was conducted at 120 health facilities in Uttar Pradesh, India. These sites were selected from a larger pool of 284 sites that met eligibility criteria, namely public-sector primary and community health facilities with delivery services 24 hours a day, 7 days a week; delivery load >1000 births per year, 3+ birth attendant staff, absence of ongoing research or other interventions that could confound trial results; and willingness of administrative and clinical leaders to participate. Women were eligible for enrollment if they presented for childbirth at study facilities and ineligible if delivered outside the facility, were referred from another facility, or admitted for abortion services. All eligible women were approached for enrollment prior to their discharge through a verbal informed consent process, which was witnessed and documented, for follow-up by a call or visit between eight and 42 days post-partum, including permission to speak with their husband, their mother, or mother-in-law if they were unavailable (Fig 1). The call center and home visit staff also reconfirmed consent at the time of the phone call or home visit. If the mother was unavailable or deceased, her husband, mother, or mother-in-law was permitted to respond to the questions and verbal informed consent from the respondent was provided over the phone. If both the mother and newborn died in the facility, the case was closed and no follow-up was done. Outcomes questions concerned maternal and neonatal mortality, five maternal morbidities, two critical interventions, and whether the mother or baby returned to the health facility given any complications (Table 1) [21]. In order to establish the timing of any adverse outcomes, respondents were asked whether each adverse outcome occurred “at any time after delivery until now.” If the respondent answered “yes,” she was asked to specify whether the adverse outcome occurred within seven days after delivery. To respect personal and cultural norms, all call center staff were female.

Three attempts were made to contact each respondent directly by phone (for whom a phone number was available in the facility register) and any respondent who was not contacted successfully by 21 days post-partum (at the latest) received a “field worker assisted call.” During a field worker assisted call, a field worker traveled to the home, confirmed consent, handed the respondent a mobile phone that was pre-connected to the call center, and stepped away to provide privacy. Due to safety concerns, the majority of field workers were male. The female call-center employee conducted the outcomes assessment. At respondent homes with no cellular network coverage, the field worker conducted the outcomes assessment face-to-face using the same script as the call center (Fig 1). Relationships were established with a vast network of community health workers throughout UP, known as Accredited Social Health Activists (ASHAs), to facilitate locating contact details for cases with incorrect or missing phone numbers or otherwise unreachable.

### Staff training and pilot testing

Call center staff underwent a comprehensive eight-day training focused on the outcomes assessment protocol, research ethics, interview techniques, adherence to the standardized script, use of the dashboard, and the DQA process. In September 2014, after the call center protocol was successfully tested for feasibility with patients discharged from three pilot sites, it was established as the primary outcomes data collection method for the BetterBirth Trial.

As part of the implementation of the call center, we trained call center staff and supervisors for appropriate sympathetic responses in case of a death or morbidity. In case of a health problem discovered on the phone, the call center staff referred the woman/newborn to the health facility or nearby clinic/doctor. We established a phone hotline where if there were problems, families could call and report concerns through Community Empowerment Lab, Lucknow, India. We reviewed the logs of those calls to ensure appropriate follow ups were made. While we tried to find appropriate community-based counseling centers across the state, they were found to be non-functional.

### Data quality assurance protocols

#### Completeness, effectiveness, and efficiency

Completeness of data entry was defined as no missing data; this was ensured by building constraints into the data-entry application to prevent inappropriately missing data. Effectiveness of the call center was defined as the proportion of cases with outcomes determined by telephone. Efficiency of the call center was defined as the number of call attempts and days required to determine presence or absence of any outcome.

#### Double coding procedure to assess accuracy

All calls were recorded; supervisors double-coded 1.8% (2,738/154,505) of closed cases. The accuracy of the data, assessed by comparing the call center staff results to the supervisors’ coding (the gold standard), was defined as a match between staff and supervisor. An equal number of calls with and without adverse outcomes were assessed, and the supervisor was blinded to all caller-entered data. Results of DQA, including caller accuracy rates and error trends for each question, were presented in daily reports. Caller errors were addressed through routine supportive supervision.

All newly hired callers underwent an intensive initial phase (designed to last 6–8 weeks), in which each caller was required to achieve perfect accuracy on four sets of 10 consecutive calls. Each set of 10 calls included five with identified outcomes and five without identified outcomes. During each quarter of the remainder of her employment (maintenance phase), each caller was required to achieve perfect accuracy on one set of ten consecutive calls. At any time, if a caller achieved <100% accuracy on a set of ten consecutive calls, the assessment was repeated on a different set of calls following supportive supervision.

#### Call center validation procedure

A subset of cases closed by the call center (N = 1,475) received a field worker assisted validation call within seven days of case closure to further validate the call center’s ability to determine the presence or absence of adverse outcomes. Response data from the first and second calls were compared for consistency.

### Sample size, data analysis, & ethics

Descriptive statistics were calculated for variables related to effectiveness, efficiency, and accuracy of the call center. Cost information was calculated from budget expenditures for infrastructure and ongoing monthly costs were estimated using cost data from May 2016, a period representative of the trial at full operation.

For the validation procedure, inconsistency, the outcome of interest, was defined whether the response to any question on the field worker assisted validation call differed from the response given on the first call (set as the gold standard). The sample size was chosen to achieve a 95% confidence interval (CI) for the percent inconsistency between the first and second call that was at most 4% (e.g., 15%+/-2%), which required a minimum of 613 cases that had adverse outcomes identified during the first call and a minimum of 613 cases with no adverse outcomes identified in the first call. Unadjusted relative risks, accounting for clustering within site, using generalized estimating equations [35], were calculated for potential predictors associated with inconsistency using univariable log-binomial regression. Since ‘respondent type’ on first call and ‘whether the respondent changed between first and second call’ are highly collinear, we *a priori* chose to use the latter in the multivariable regression analyses. The final model estimated relative risk (RR) and 95% CI for predictors of inconsistency using a multivariable relative risk regression [36], clustering by site. All statistical analyses were done using SAS 9.4 (SAS Institute, Cary, NC).

The study protocol, including consent process and sub-analyses, were reviewed and approved as part of the BetterBirth Trial study protocol which was approved by all participating institutions: Community Empowerment Lab (CEL) Ethics Review Committee formerly Lucknow Ethics Committee, Jawaharlal Nehru Medical College Ethical Review Committee, Institutional Review Board of the Harvard T.H. Chan School of Public Health, Population Services International Research Ethics Board, and the Ethical Review Committee of the World Health Organization. The Indian Council of Medical Research also approved the study. The protocol was reviewed and reapproved on an annual basis. The trial is registered at ClinicalTrials.gov (Identifier: NCT02148952).

## Results

### Effectiveness and efficiency of call center

Between February 2015 and January 2017, the call center successfully followed-up 98.0% (n = 154,494) of eligible cases; 87.9% of the outcomes were recorded by phone alone (Table 2). The remaining 12.1% of cases required a field worker assisted call, often due to missing phone numbers in the patient record. Additional cases were closed by a field worker alone (1.7%, n = 2,745) due to lack of network connectivity. A small proportion of cases enrolled in the trial retracted consent (0.1%, n = 94) or were lost-to-follow-up (0.3%, n = 450). Loss to follow-up most commonly occurred when women moved out of the study catchment area or contact details were inaccurate. Participant characteristics have been described elsewhere [22].There are no clinically significant differences in measured demographic indicators within or across the three groups who were enrolled but whose outcomes were not collected (the patients who were lost to follow-up, those whose outcomes were assessed by a field worker only, and those who retracted consent) as compared with study participants whose outcomes were assessed by the call center (S1 Table). Significant differences (p<0.001) in rates of early neonatal mortality were reported for cases closed by the call center only (4.4%, 95% CI 4.1%-4.7%, n = 6005/135,698), compared with field worked assisted calls (6.7%, 95% CI 6.1%-7.4%, n = 1256/18,718), and field worker only (7.3%, 95% CI 5.9%-9.1%, n = 200/2,728).

**Table 2:** Effectiveness, efficiency, cost, and accuracy of call center (3 Feb ‘15–7 Jan ‘17).

Call center effectiveness	n (%)
Total number of cases enrolled	157,689 (100)
Closed by field worker only	2,745 (1.7) *
Lost to follow-up	450 (0.2) *
Retracted consent (on call or at home visit)	94 (0.1)
Total closed by call center	154,494 (98.0) *
Closed by call only	135,767 (87.9) ^
Closed by field worker assisted call	18,727 (12.1) ^
**Call center efficiency**	**n**
Callers	26
Supervisors	6
Managers	1
	**median (IQR)**
Calls closed per day, per caller	17 (12–21)
	**median (IQR)**
Days to determine outcome	3 (1–5)
Calls to determine outcome	1 (1–2)
**Field worker efficiency**	**median (IQR)**
Days to determine outcome	7 (3–13)
Visits to determine outcome	1 (1–1)
**Cost of follow-up methods**	**USD**
**One-time establishment and infrastructural costs**	
Call center (laptops, desktops, phones, headsets, call recorder)	16,015.45
Field worker home visitation (tablets, smartphones, power banks)	16,563.29
**Operational costs, per month (referenced from May 2016)**	
Call center (telephone bill, caller and supervisor salaries, rent and utilities)	11,947.25
Field worker home visitation (rent and utilities, field worker and supervisor salaries, transportation costs)	62,176.07
Field worker assisted calling (combined operational costs for call center and field worker home visitation)	74,123.32
**Operational cost per case closed, May 2016 (n = 10,979)**	
Call center cost (actual)	1.09
If closed by field worker only	5.66
If closed by field worker assisted call	6.75
**Call center DQA accuracy**	**n (%)**
Questionnaire-level accuracy** (N = 2,794)	2,738 (98.0)
Question-level accuracy^^ (N = 81,026)	80,095 (99.9)

* Percent is calculated out of "Total number of cases enrolled"

^ Percent is calculated out of "Total cases closed by call center"

** Questionnaire-level accuracy: total questionnaires with zero errors

^^ Question-level accuracy: total questions with zero errors

**Fig 2:**
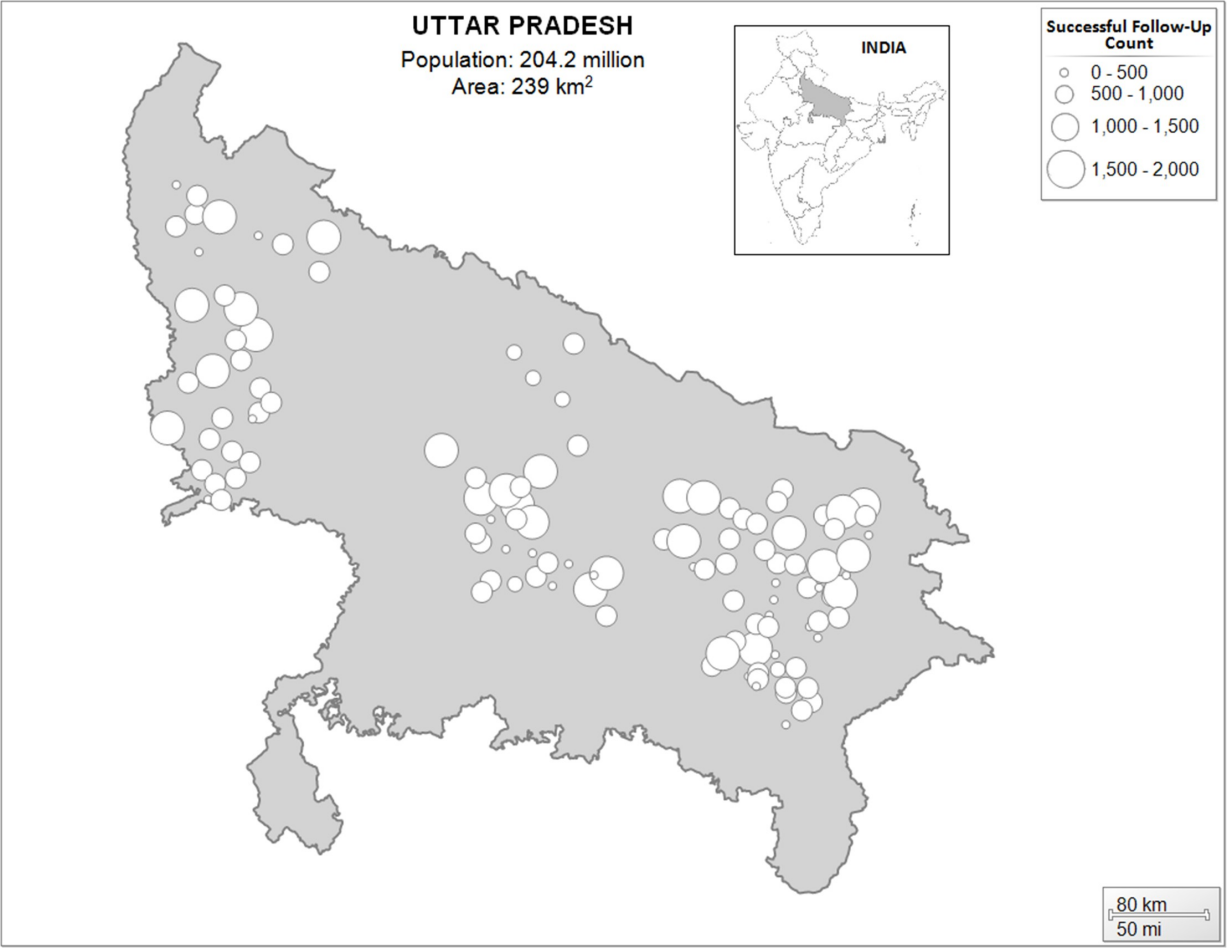
Geographic distribution and volume of cases successfully followed-up by call center, 3 Feb 2015–7 Jan 2017.

The call center increased from three callers and one supervisor to 26 callers, six supervisors, and one manager as the number of cases requiring follow-up increased. The median duration from the start of the follow-up period to case closure was 3 days (IQR 1–5), and a median of 1 call (IQR 1–2) was required to close a case (Table 2). Cases closed by the call center were distributed over a large geographic area (Fig 2).

### Cost of the call center

The operational cost per case closed by the call center was approximately 1 dollar (1.09 USD). If only field workers had been used and closed all cases, the cost per case closed would have been approximately five times greater (5.66 USD). If all cases were closed by field worker assisted calls, the cost per case closed would be approximately six times higher than the call center alone (6.75 USD) (Table 2).

### Accuracy of data

Of the 2,794 calls that were double-coded, the rate of perfect accuracy (questionnaires with zero errors across all questions) was 98.0% (Table 2). The rate of accuracy for individual questions (n = 81,206) was even higher at 99.9%.

### Validation of call center

The validated sample included 1,475 cases, 794 with adverse outcomes identified on the first call and 681 without adverse outcomes identified on the first call. In the validated sample, the first call most often reached the mother within two to five days of eligibility for follow-up, and of the 1,475 validated cases, 93.7% demonstrated perfect consistency between the first and second calls.

We had a total of 93 follow-up calls with inconsistency, and 116 inconsistent responses to individual questions. Among inconsistent responses, two questions comprised the majority on adverse outcomes in the seven days post-delivery: presence of fever (n = 37/116, 31.9%) and occurrence of excessive bleeding (n = 30/116, 25.9%). Other questions for which inconsistent responses were given included: mother returning to the facility (n = 21/116, 18.1%), baby returning to the facility (n = 18/116, 15.5%), baby death (n = 6/116, 5.2%), and loss of consciousness (n = 4/116, 3.5%). All kappa coefficients were >0.89. The respondent on the first call was most often the mother. The median number of days between the first and second call was 8 (IQR 4–15); the majority (87.1%) had the same respondent for both calls (Table 3).

**Table 3:** Bivariate analyses of predictors of inconsistent call center responses.

					Risk of Inconsistency		
	N (%)	Median (IQR)	Consistent (%)	Inconsistent (%)	RR*	95% CI*	p-Value
Total outcomes surveys validated	1,475 (100)	-	1,382 (93.7)	93 (6.3)			
Days until first call		3 (2–5)			1.01	0.94–1.07	0.865
Respondent type, first call							
Mother	1,347 (91.3)		1,267 (94.1)	80 (5.9)	1	-	
Husband	105 (7.1)		97 (92.4)	8 (7.6)	1.28	0.79–2.07	0.310
Other	23 (1.6)		18 (78.3)	5 (21.7)	3.66	1.80–7.43	<0.001
Identified outcome on first call							
No identified outcome	681 (46.2)		667 (97.9)	14 (2.1)	1	-	
At least 1 identified outcome	794 (53.8)		715 (90.1)	79 (9.9)	4.84	2.78–8.43	<0.001
Days between first call and second call		8 (4–15)			1.03	1.00–1.05	0.071
Days 0–14	1,097 (74.4)		1,041 (94.9)	56 (5.1)			
Days 15+	378 (25.6)		341 (90.2)	37 (9.8)	1.92	1.18–3.12	0.001
Respondent change between first and second call^‡^							
Respondent same	1,282 (87.1)		1,212 (94.5)	70 (5.5)	1	-	
Respondent different	190 (12.9)		168 (88.4)	22 (11.6)	2.12	1.42–3.16	<0.001

* RR indicates relative risk; CI, confidence interval

^‡^ If "other" was the respondent type on both the first and second call they were dropped from this analysis since it was not possible to determine if the respondent was the same between calls (n = 3)

#### Predictors of inconsistency

When an adverse outcome was reported on the first call, the rate of inconsistency with the second call was almost five times higher than the rate of inconsistency between the two calls when no adverse outcome was reported in the first call (9.9 versus 2.1). When the first call reached the mother or the mother’s husband, inconsistency was much lower than when the first call reached anyone else (no matter whom the second call reached). Whenever the respondent to the second call differed from the first, the rate of inconsistency was approximately double the rate of inconsistency compared to those with the same respondent on both calls (Table 3).

#### Multivariable regression results

The final multivariable regression model revealed three major factors associated with significantly increased risk of inconsistency. Identification of an adverse event on the first call was the strongest predictor of inconsistency (RR = 4.78, 95% CI [4.22–5.34]). When the respondent changed between the first and second call, the risk of inconsistency was twice as high compared to when the respondent was the same (RR = 2.03, 95%CI [1.70–2.36]). Additionally, second calls which occurred >15 days from the first call had a greater risk of inconsistency (RR = 1.85, 95% CI [1.39–2.31]) than second calls conducted within fourteen days of the first call (Table 4).

**Table 4:** Multivariable analysis for the prediction of inconsistency.

	Risk of Inconsistency		
	RR*	95% CI*	p-Value
Days until first call (continuous)	1.00	0.94–1.05	0.873
Respondent change between first and second call^‡^			
Respondent same	1		
Respondent different	2.03	1.70–2.36	<0.001
Identified outcome on first call			
No identified outcome	1		
At least 1 identified outcome	4.78	4.22–5.34	<0.001
Days between first call and second call			
Days 0–14	1		
Days 15+	1.85	1.39–2.31	0.017

* RR indicates relative risk; CI, confidence interval

^‡^ If "other" was the respondent type on both the first and second call they were dropped from this model since it was not possible to determine if the respondent was the same between calls (n = 3)

## Discussion

This study describes the successful implementation and validation of a call center in ascertaining patient-reported post-discharge outcomes for mothers and their babies in a low-resource, geographically vast setting. These findings have the potential to influence how health systems, programs, and research trials gather post-discharge outcomes on patients in regions where cellular coverage is high to inform efforts to improve healthcare access, quality and outcomes for mothers and their newborns. Additional work to evaluate the call center method through clinical validation of patient outcomes and the development of simplified assessments/surveys for patient reported outcomes is necessary.

Acceptable rates of follow-up in epidemiological studies are generally agreed to be 60–80%, however the call center successfully closed 98.0% of eligible cases (87.9% by call center alone), which we believe is largely attributable to three factors [37]. First, mobile coverage in the study area is high. In February 2016, the Telecom Regulatory Authority of India reported a total of 147,772,742 wireless cellular subscribers throughout Uttar Pradesh [38]. Cell phone coverage is estimated to be 74.0% [39] and is likely much higher. While there are still populations with no access to mobile phones or cellular networks, relying on field workers to close the ‘harder to reach’ cases proved to be an effective alternative method. Second, depending on facility-based study staff to collect consenting mothers’ contact information and automating the link between the facilities and the call center ensured that the call center dashboard was routinely updated with the latest information on mothers eligible for follow-up. Third, call center staff relied on ASHAs to locate mothers and their families when the mothers were unreachable or the contact number given at the facility was missing or incorrect. In these cases, the community-based ASHAs arranged for calls, or field workers were sent to the home to conduct a field worker assisted call.

Despite the eight-fold growth of call center staff over the course of the study to accommodate increasing study enrollment, caller accuracy rates remained consistently high. Regular testing and support of callers’ work quality was integrated into call center operations through the supportive components of the DQA protocol. Real-time reports generated by the call center’s dashboard (featuring error incidence by question) enabled supervisors to provide supportive coaching to the callers. Given the phase-specific goals for each caller, performance was regularly tested and integrated into call center operations. In addition, we adopted core concepts of supportive supervision. Accuracy was celebrated, and errors were discussed between caller and supervisor. Sources of error were identified from call recordings, and improvement strategies were identified.

Of the total validation sample, 93.7% of cases were perfectly consistent. Given limited published data on the validity of call centers for ascertaining patient-reported outcomes in Uttar Pradesh and similar settings, these findings are novel and promising. Several factors contributed to the 6.3% of cases, which had at least one inconsistent response between the first and second calls of the validation procedure. First, allowing for surrogate respondents on the mother’s behalf resulted in higher rates of inconsistency. Not surprisingly, our data show that the mother is the preferred respondent when answering questions about her own and her baby’s health. However, the range of possible barriers to reaching a mother (e.g. the mother has died, she prefers another family member to answer on her behalf) may necessitate seeking alternative sources of information. There is considerable evidence demonstrating sole reliance on the birth mother to respond about neonatal mortality may significantly underestimate baby deaths due to the positive correlation between maternal and neonatal deaths [40]. Questionnaires must be designed to maximize response reliability from multiple respondents. Additionally, we attempted to call respondents a second time within seven days of the first call during validation exercises; however, this timing was not always possible due to the high volume of cases. Cases with delayed validation were almost twice as likely to demonstrate an inconsistent response on the second call. Call-center protocols and validation procedures should consider the timing of calls to limit recall bias.

Based on the count of cases closed by the call center alone in May 2016 (n = 10,979), we extrapolated the cost if these cases had been closed by a field visit and by a field worker assisted call. Contacting mothers only by phone was the least costly follow-up method at $1.09 per case closed, followed by field visits and field worker assisted calls, costing $5.66 and $6.75, respectively. In addition to cost differences between the three modes of follow-up, each mode required differing time duration to case closure. Though each method only took one attempt, the call center closed cases in a median of three days, whereas it took a median of six days for field workers to close a case. We also compared our methods with population health surveys, which are commonly used throughout the world to ascertain maternal and neonatal health data, and are estimated to cost between $7.00 and $104.00 per survey visit [41, 42]. Based on the differential costs of the three methods used in this study as well as comparisons with independent population health surveys, telephonic follow-up may be a less costly option. Additionally, accurate recall is a concern with annual population health surveys that target the immediate post-natal period, therefore the call center’s validated and timely follow-up suggests cost-effectiveness in terms of data capture [43].

We did find a small but significant difference in the rates of reported early neonatal mortality ascertained for cases reached by the call center alone versus those for whom a field visit was required. The rates were higher for cases unable to be reached by the call center alone. A number of factors may have contributed to this difference in early neonatal mortality rates which could include higher prevalence risk factors for early neonatal mortality among families without cell phone access, cellular coverage, or reluctance to report deaths by phone. Work is planned to explore the underlying causes of these differences. We recommend that implementers collect preliminary data to measure whether outcomes are similar among participants with and without cellular phone or network access. For example, home visitation may be necessary if lack of cellular access is found to be associated risk factors related to the health outcome of interest. If similar, call center implementers may consider focusing efforts and resources on collecting outcomes directly by phone.

In settings where mobile coverage is high, implementing telephonic follow-up may be a reasonable alternative to solely relying on home visitation for data collection, which historically has been the standard practice for post-delivery outcomes assessment. There are considerable limitations to home visitation for large-scale research and implementation projects, especially among geographically dispersed populations. First, field workers can only target a limited geographic area and visit a small number of participants per day, potentially resulting in longer time periods between discharge and follow-up, which in our experience is associated with lower rates of consistency. Second, supervision to assess data quality is logistically challenging and resource intensive, requiring the supervisor to either observe or revisit the interview. Third, in many regions where independent travel by female field workers is not feasible due to safety concerns, the necessary employment of male field workers to ascertain responses to questions about neonatal and maternal complications (a personally and culturally sensitive topic) may compromise the accuracy and reliability of respondents’ answers. In settings where cellular coverage is low and safety is a concern for female field workers, relying on village-based female community health workers to collect maternal and early neonatal outcomes may be more reliable and cost-effective [38]. Telephone-based outcomes assessment is not meant to replace existing home visitation programs. This approach, however, could support the growing practice of capturing maternal and neonatal health status after discharge and outcomes auditing, facilitating more timely data capture than population health surveys and better designed for ongoing program improvement [44, 45].

There are a few limitations to this study. We relied on patient or family member report for our post-discharge outcomes and were not able to conduct a clinical verification. The accuracy of maternal-reported early neonatal complications can be variable due the type and severity of events, a mother’s medical knowledge, and her level of education. Research also suggests that parental stress and grief following an infant death can significantly affect their ability to recall details related to the events. However, the use of patient reported outcomes to capture adverse events after discharge is a growing standard in many fields, offers insights into patient’s experiences and perceptions, but more work is needed to better understand the limitations [46–54]. In order to make the outcomes questionnaire understandable, feasible and acceptable, we had to modify the “near-miss” questions. Since we did not clinically validate the outcomes, we did not validate the adaptation of the questions. There were also limitations in our validation process. We were unable to use the existing gold standard of a field worker home visit to repeat the questionnaire because of safety concerns for female field workers and the reluctance we observed during piloting in reporting some of the morbidities to a male interviewer made this unreliable.

## Conclusions

Our findings, especially given the paucity of neonatal outcomes measurement post-facility discharge in the community setting [3, 55], demonstrate the promising potential for the use of call centers to collect post-discharge information. Our follow-up protocol, combining the efforts of a call center and field workers, achieved an extremely high follow-up rate of 99.7%, resulting in minimal loss-to-follow-up. The call center closed an impressive 98.0% of eligible cases, with the vast majority closed by a single call. Given the relatively low incidence of adverse neonatal and maternal outcomes at the population level, follow-up modalities with limited loss-to-follow-up are essential to ensure accurate reporting and to limit underreporting of events [56].

Our call center was highly effective in ascertaining patient-reported outcomes by successfully following up with the large majority of cases in an under-resourced and geographically vast setting. Although clinical validation is needed in future work, the call center demonstrated high accuracy and validity as a follow-up method. Remote assessment of patient outcomes by phone offers an exciting and low cost approach for rapidly and reliably assessing outcomes in parts of the world where mobile coverage is high.

## Supporting information

S1 Table Demographic characteristics of respondents by follow up type for post-discharge health outcomes assessment in Uttar Pradesh, India. (DOCX)
