# Supplementary material for: Evaluation of a call center to assess post-discharge maternal and early neonatal outcomes of facility-based childbirth in Uttar Pradesh, India
Source: PLoS One. 2018 Nov 27;13(11):e0207987. doi: 10.1371/journal.pone.0207987 (PMC6258538; doi:10.1371/journal.pone.0207987)
Supplement: S1 Table — (DOCX) [file pone.0207987.s001.docx]

**S1 Table. Demographic characteristics of respondents by follow up type for post-discharge health outcomes assessment in Uttar Pradesh, India**

|  |  | **Call Center Only** | | | | | **Field Worker Assisted Call** | | | | | **Field Worker Only** | | | | | **Lost to Follow-up** | | | | |
| --- | --- | --- | --- | --- | --- | --- | --- | --- | --- | --- | --- | --- | --- | --- | --- | --- | --- | --- | --- | --- | --- |
|  | **Overall p-Value for trend** | **n** | **N** | **% or mean** | **Lower 95% CI** | **Upper 95% CI** | **n** | **N** | **% or mean** | **Lower 95% CI** | **Upper 95% CI** | **n** | **N** | **% or mean** | **Lower 95% CI** | **Upper 95% CI** | **n** | **N** | **% or mean** | **Lower 95% CI** | **Upper 95% CI** |
| N |  | 135767 | 157689 | 86.10% |  |  | 18727 | 157689 | 11.88% |  |  | 2745 | 157689 | 1.74% |  |  | 450 | 157689 | 0.29% |  |  |
| Mean age (95% CI) - year | <0.001 | 135140 |  | 25.63 | 25.49 | 25.77 | 18491 |  | 25.83 | 25.67 | 26.00 | 2717 |  | 25.97 | 25.74 | 26.20 | 443 |  | 25.87 | 25.49 | 26.26 |
| Delivery facility level | 0.004 |  |  |  |  |  |  |  |  |  |  |  |  |  |  |  |  |  |  |  |  |
| Primary health center |  | 50683 | 135767 | 37.33% | 28.18% | 46.48% | 6112 | 18727 | 32.64% | 23.48% | 41.79% | 899 | 2745 | 32.75% | 19.61% | 45.89% | 102 | 450 | 22.67% | 9.45% | 35.88% |
| Community health center |  | 63651 | 135767 | 46.88% | 37.43% | 56.34% | 10149 | 18727 | 54.19% | 44.08% | 64.31% | 1313 | 2745 | 47.83% | 34.18% | 61.49% | 273 | 450 | 60.67% | 42.59% | 78.74% |
| First referral unit |  | 21433 | 135767 | 15.79% | 8.78% | 22.79% | 2466 | 18727 | 13.17% | 6.62% | 19.72% | 533 | 2745 | 19.42% | 7.85% | 30.98% | 75 | 450 | 16.67% | 4.73% | 28.60% |
| Mean previous pregnancies (95% CI) | <.0001 | 112155 |  | 2.35 | 2.30 | 2.39 | 14938 |  | 2.42 | 2.36 | 2.47 | 2214 |  | 2.50 | 2.41 | 2.58 | 366 |  | 2.34 | 2.14 | 2.54 |
| No. of offspring - no. (%) | 0.53 |  |  |  |  |  |  |  |  |  |  |  |  |  |  |  |  |  |  |  |  |
| Singleton |  | 134870 | 135743 | 99.36% | 99.31% | 99.41% | 18580 | 18727 | 99.22% | 99.08% | 99.35% | 2726 | 2739 | 99.53% | 99.26% | 99.79% | 379 | 382 | 99.21% | 98.45% | 99.98% |
| Sets of twins |  | 863 | 135743 | 0.64% | 0.58% | 0.69% | 147 | 18727 | 0.79% | 0.65% | 0.92% | 13 | 2739 | 0.47% | 0.21% | 0.74% | 3 | 382 | 0.79% | 0.02% | 1.55% |
| Set of triplets |  | 10 | 135743 | 0.01% | 0.00% | 0.01% | 0 | 18727 | 0% | - | - | 0 | 2739 | 0% | - | - | 0 | 382 | 0% | - | - |
| Providers attending delivery - no. (%) |  |  |  |  |  |  |  |  |  |  |  |  |  |  |  |  |  |  |  |  |  |
| Doctor | 0.007 | 19745 | 135767 | 14.54% | 8.88% | 20.21% | 2274 | 18727 | 12.14% | 7.20% | 17.08% | 223 | 2745 | 8.12% | 3.87% | 12.38% | 114 | 450 | 25.33% | 4.07% | 46.60% |
| Nurse | <.0001 | 112040 | 135767 | 82.52% | 78.41% | 86.64% | 14643 | 18727 | 78.19% | 73.32% | 83.07% | 2193 | 2745 | 79.89% | 73.27% | 86.51% | 293 | 450 | 65.11% | 50.81% | 79.41% |
| Auxiliary nurse midwife | 0.32 | 25438 | 135767 | 18.74% | 14.21% | 23.27% | 3592 | 18727 | 19.18% | 14.00% | 24.36% | 445 | 2745 | 16.21% | 11.10% | 21.32% | 135 | 450 | 30% | 10.09% | 49.91% |
| Other | 0.03 | 7837 | 135767 | 5.77% | 1.87% | 9.67% | 999 | 18727 | 5.33% | 1.83% | 8.84% | 290 | 2745 | 10.56% | 2.06% | 19.06% | 14 | 450 | 3.11% | 0% | 7.22% |
